# GL-YOLO-Lite: A Novel Lightweight Fallen Person Detection Model

**DOI:** 10.3390/e25040587

**Published:** 2023-03-29

**Authors:** Yuan Dai, Weiming Liu

**Affiliations:** School of Civil Engineering and Transportation, South China University of Technology, Guangzhou 510641, China; ctdaiyuan@mail.scut.edu.cn

**Keywords:** fallen person detection, deep learning, computer vision, object detection, lightweight neural networks, binary cross-entropy

## Abstract

The detection of a fallen person (FPD) is a crucial task in guaranteeing individual safety. Although deep-learning models have shown potential in addressing this challenge, they face several obstacles, such as the inadequate utilization of global contextual information, poor feature extraction, and substantial computational requirements. These limitations have led to low detection accuracy, poor generalization, and slow inference speeds. To overcome these challenges, the present study proposed a new lightweight detection model named Global and Local You-Only-Look-Once Lite (GL-YOLO-Lite), which integrates both global and local contextual information by incorporating transformer and attention modules into the popular object-detection framework YOLOv5. Specifically, a stem module replaced the original inefficient focus module, and rep modules with re-parameterization technology were introduced. Furthermore, a lightweight detection head was developed to reduce the number of redundant channels in the model. Finally, we constructed a large-scale, well-formatted FPD dataset (FPDD). The proposed model employed a binary cross-entropy (BCE) function to calculate the classification and confidence losses. An experimental evaluation of the FPDD and Pascal VOC dataset demonstrated that GL-YOLO-Lite outperformed other state-of-the-art models with significant margins, achieving 2.4–18.9 mean average precision (mAP) on FPDD and 1.8–23.3 on the Pascal VOC dataset. Moreover, GL-YOLO-Lite maintained a real-time processing speed of 56.82 frames per second (FPS) on a Titan Xp and 16.45 FPS on a HiSilicon Kirin 980, demonstrating its effectiveness in real-world scenarios.

## 1. Introduction

A report by the World Health Organization (WHO) [1] highlighted falls as the main cause of health concerns among seniors, with an alarming 4–15% of falls resulting in serious injury and a significant 23–40% of elderly fatalities being attributed to falls. Given the severity of the consequences associated with falls in the elderly population, it is imperative that proactive measures are taken to detect these incidents. Accordingly, there is a pressing need for algorithms capable of accurately recognizing and assessing human falls.

Fallen person detection (FPD) technology has been categorized into three primary implementation methods: scene perception, wearable devices, and visual-based approaches. Scene-perception-based FPD algorithms [2,3] have utilized non-video sensors, such as pressure and acoustic sensors, placed around pedestrian walking areas to capture human body feature information. This method suffers from limited applicability due to its high cost and susceptibility to environmental interference, such as noise, which leads to high detection error rates. Wearable-device-based FPD research [4,5] has typically embedded sensors in user-worn devices, such as smart bracelets and mobile phones. However, wearing various sensors over long periods has caused discomfort for some users, and complex activities have been misinterpreted as falls. Additionally, the size and the portability of these devices present their own challenges, including battery life, maintaining connectivity, and data transmission.Visual-information-based FPD technology [6,7] collects image or video data through fixed image or video acquisition equipment at the detection site, and then it identifies the human body using traditional image-processing technology or deep-learning-based methods. The accuracy of FPD systems has improved significantly [6,8,9,10,11] due to the emergence of deep learning and neural network models in computer vision. However, limitations still exist within the deep learning paradigm, including the inadequate utilization of global contextual information [12,13], sub-optimal feature extraction [10], and large floating-point operations (FLOPs) and model parameters [11,14]. These shortcomings have resulted in poor overall accuracy, limited robustness and generalization, and slow detection speeds.

This study proposed a novel lightweight detection model, Global and Local You-Only-Look-Once Lite (GL-YOLO-Lite), to address the challenges of low detection accuracy and slow detection speeds associated with current deep-learning object-detection algorithms. GL-YOLO-Lite leveraged transformer modules [15] and an attention module [16] to capture the global contextual information and employed convolutional layers to extract local information, thus enhancing feature representation. Furthermore, GL-YOLO-Lite replaced the original inefficient focus module in YOLOv5 [17] with a stem module [18] that comprised standard convolutional units and utilized rep modules [19] with re-parameterization technology, enabling the model to employ both multi-branch and single-path methods.

Additionally, we created and labeled a large-scale, well-structured FPD dataset (FPDD) by collecting online images and taking photos. By conducting comparison experiments on the FPDD and the Pascal VOC dataset [20,21], we demonstrated that GL-YOLO-Lite had superior performance. According to the mean average precision (mAP@0.5), frames per second (FPS), and the technique for order preference by similarity to an ideal solution (TOPSIS) metrics, GL-YOLO-Lite outperformed other state-of-the-art lightweight models. We present a streamlined comparison of the highlights and the limitations of various FPD technologies in Table 1.

To sum up, the contributions of this study were four-fold, as follows:

- Drawing from YOLOv5, GL-YOLO-Lite introduced transformer and attention modules, which were capable of capturing long-range dependencies and enabled the model to better integrate global and local features. This improved the detection accuracy significantly.
- We improved GL-YOLO-Lite by using a stem module instead of the focus module, adding rep blocks for re-parameterization, and designing a light-weight detection head. These changes made GL-YOLO-Lite faster.
- We created and labeled a large-scale, well-structured dataset, FPDD, by collecting online images and taking photos. This filled the gap in existing FPD datasets.
- The efficacy of the proposed GL-YOLO-Lite was validated through experiments on the FPDD and the Pascal VOC dataset. Our results showed that GL-YOLO-Lite had a 2.4–18.9 mAP improvement over the state-of-the-art methods on FPDD and a 1.8–23.3 mAP improvement on the Pascal VOC dataset. Furthermore, our model achieved top-tier TOPSIS scores.

## 2. Related Works

### 2.1. Fallen Person Detection Based on Scene Perception

The application of fall detection technology based on scene perception involves the deployment of a variety of sensors, including vibration, sound, pressure, infrared, and WiFi sensors, to monitor and collect human-specific data in and around objects, such as walls, floors, and beds. The different characteristics of a target person in various states are subsequently used to determine whether a fall has occurred. Notably, Yazar et al. [22] employed two vibration sensors and two infrared sensors to collect data and analyze the movement state of a person. Luo et al. [23] developed a large-scale pressure pad and indoor motion detection device and identified falls using a decision-tree algorithm. Mazurek et al. [24] utilized an infrared depth sensor to acquire the position information of a person and applied a Bayesian algorithm to determine whether a fall had occurred. Wang et al. [25] proposed a human behavior recognition method based on an infrared sensor array that classified human motion using temperature information obtained by the sensor. Zhang et al. [26] proposed a fall detection method based on the Doppler effect of ultrasound. This method relied on the frequency offset of reflected ultrasound to determine whether a person’s motion reflected a fall.

Despite these achievements in scene-perception-based fall detection technology, significant challenges remain. First, this technology is limited to relatively stable indoor environments to minimize environmental interference and ensure accurate fall recognition, rendering it unsuitable for more complex scenarios. Second, it is highly susceptible to external environmental influences, with weak anti-interference and a high error rate in detection. Lastly, the equipment used for collecting information based on scene perception is often expensive and requires multi-sensor-fusion processing, resulting in higher use costs that may not be feasible.

### 2.2. Fallen Person Detection Based on Wearable Devices

Fall detection technology based on wearable devices involves integrating sensors into devices worn by users, such as smart bracelets or mobile phones, to collect the relevant data. The data collected by these sensors are then transmitted to a fall determination model for the purpose of detecting falls. Peng et al.’s approach [27] involved using a simple threshold for body acceleration and angular velocity values collected by a belt, followed by further analysis of the data through algorithms in the main controller’s processor. Similarly, Rakhman et al. [28] utilized the high-precision three-axis accelerometers and gyroscopes built into modern smartphones to identify fall actions by selecting an appropriate threshold. Shahiduzzaman [29] set a threshold to determine whether a fall had occurred based on motion signals collected by an accelerometer and heart-rate signals obtained by a heart rate variability (HRV) sensor. Jefiza et al. [4] proposed a back-propagation fall detection algorithm based on accelerometer and gyroscope data. This approach constructed a 10-dimensional motion feature from the data acquired by a three-axis accelerometer and gyroscope and input it into a back-propagation neural network to obtain a fall detection model.

Despite their increasing popularity, wearable-device-based fall detection technology has limitations. Firstly, prolonged use of multiple sensors has significantly affected user comfort and has also led to the misidentification of complex human activities, such as falls. Secondly, the limited size and portability of these wearable devices has been a major barrier to their adoption. Finally, efficient connectivity and data transmission must be maintained at all times, which imposes higher demands on the hardware and software.

### 2.3. Fallen Person Detection Based on Visual Information

The installation of surveillance devices in pedestrian areas to collect real-time video images has provided a foundation for fall detection technology based on visual information. Traditional image-processing and deep-learning-based computer vision technologies can then be employed to identify, detect, and determine the position and movement of a body and whether a fall has occurred. For example, Cui et al. [30] divided the human body into multiple parts and used interpolation to extract three-dimensional coordinates associated with the key joints. They then utilized a support-vector machine (SVM) to detect human body joints and record the motion changes of the joints according to their spatial positions to determine a falling action. Similarly, Wang et al. [31] first utilized OpenPose [32] to obtain human skeleton information and then input this information into a 3D convolutional neural network (CNN) to extract spatiotemporal features and determine falling actions. Zhu [33] used YOLOv5 [17] to detect pedestrians and programmed a detection box into the DeepSort [34] algorithm to track pedestrians and obtain the temporal characteristics of human behaviors.They used a CNN to extract movement features within the tracking box, and then they employed a bidirectional long short-term memory (LSTM) algorithm based on an attention mechanism for fall detection. Despite these potential advancements, several challenges remain for the successful implementation of visual information-based falling detection technology. Firstly, the lack of a large-scale, well-formatted, and accurately labeled public dataset is a significant obstacle to deep learning-based FPD algorithms. Secondly, current FPD algorithms require improvements in recognition accuracy, model robustness, and generalization. Lastly, current deep neural networks are computationally expensive, resulting in slow detection speeds and an inability to meet real-time detection requirements.

## 3. GL-YOLO-Lite

### 3.1. Overview

**Figure 1:**
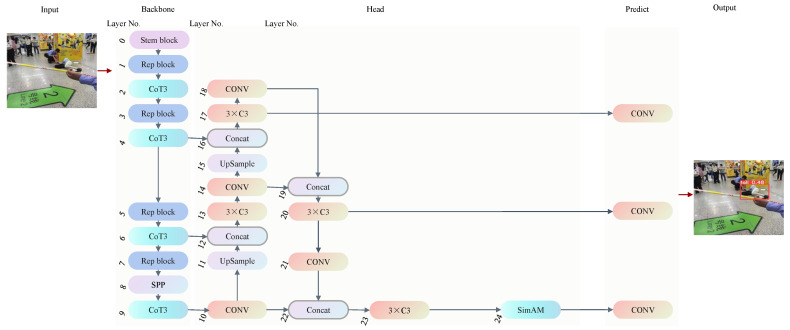
Architecture of GL-YOLO-Lite. The number of each layer is marked in black numbers.

Figure 1 illustrates the model structure of GL-YOLO-Lite, which was based on YOLOv5 [17]. To address the issue of low accuracy resulting from an inadequate use of global contextual information in the original YOLOv5, this study introduced transformer and attention modules [10,35,36]. By combining convolutional modules from the original YOLOv5 with transformer and attention modules, the model could effectively utilize both local and global contextual information. Furthermore, to improve the detection accuracy, *K*-means++ [37] was employed to generate new anchor boxes instead of *K*-means [38], which overcame the strong dependence of *K*-means on cluster-center initialization. These operations resulted in a highly accurate model, GL-YOLO. Stem modules [18] composed of standard convolutional units replaced the original focus module; rep modules [19] based on re-parameterization technology were used to improve the model’s performance and speed. The detection head of the model was optimized to further reduce the model’s FLOPs. This improvements results in the proposed GL-YOLO-Lite.

### 3.2. Loss Function in GL-YOLO-Lite

GL-YOLO-Lite utilized a binary cross-entropy (BCE) [39] function to calculate the classification and confidence losses. The BCE function was defined as follows:

(1) $$Loss\left(g,p\right)=-g\log p-\left(1-g\right)\log\left(1-p\right)=\left\{\begin{array}{cc} -\log p,g=1 & \\ -\log\left(1-p\right),g=0 \end{array}\right.$$

where *g* represents the true label, which could take on a value of 0 or 1; *p* represents the predicted probability of the positive class; and log is the natural logarithm.

### 3.3. More Accurate Anchors Generation

Recently, deep CNN-based object-detection algorithms [40,41] have made significant advances, with an anchor mechanism being widely adopted in state-of-the-art object-detection frameworks. These approaches have demonstrated remarkable performances on commonly used public datasets, such as Pascal VOC [20,21]. The existing anchor generation methods have been classified into two categories: manual and clustering, with *K*-means as one of the most commonly used clustering algorithms. YOLOv2 [42], YOLOv3 [43], and YOLOv5 have also used *K*-means clustering to generate anchors on the MS COCO dataset [44]. However, *K*-means suffers from a critical drawback, as its convergence is heavily dependent on the initialization of the cluster center. Therefore, the final result is affected by initial point selection, leading to localized optima. To address this issue, this study proposed the use of *K*-means++ to generate anchors. As compared to *K*-means, *K*-means++ selects center points that tend towards global optima, rather than localized optima. This generated higher-quality anchors and improved detection accuracy. Subsequent experiments have confirmed the efficacy of this approach.

### 3.4. Stronger Feature Extract

Convolutional neural networks (CNNs) have demonstrated superior performance in various computer-vision tasks, such as image classification [45,46] and object detection [47,48], due to their powerful visual representation learning capabilities. However, CNNs also suffer from limitations, such as a small receptive field in their convolutional layers and their inefficiency in stacking convolutional layers to increase the receptive field. This has resulted in the inadequate capture of global contextual information. In recent years, transformers [49,50,51] have been widely used in various natural language processing (NLP) tasks due to their powerful global modeling capabilities. Models such as ViT [52] and DETR [53] have also adopted a transformer structure for long-distance modeling, which could effectively utilize the global information of images. However, the self-attention structure in the original transformer only considers the interaction between a query and a key, thereby ignoring the connections between adjacent keys. As a result, transformers excel at capturing long-distance dependencies but are inadequate for utilizing local features.

This study explored the potential of combining the advantages of both CNNs and transformers to improve detection accuracy. By leveraging local features extracted by CNNs and global contextual information captured by transformers, this study aimed to enhance detection effectiveness. While attention mechanisms for modeling global contextual data have shown good performance, they have also resulted in increased computational burdens when applied to smaller networks. Therefore, the focus of this study was to investigate more effective methods for integrating transformer and attention modules into YOLOv5 for capturing global contextual information while avoiding any increases in computational demand.

#### 3.4.1. Transformer Block

**Figure 2:**
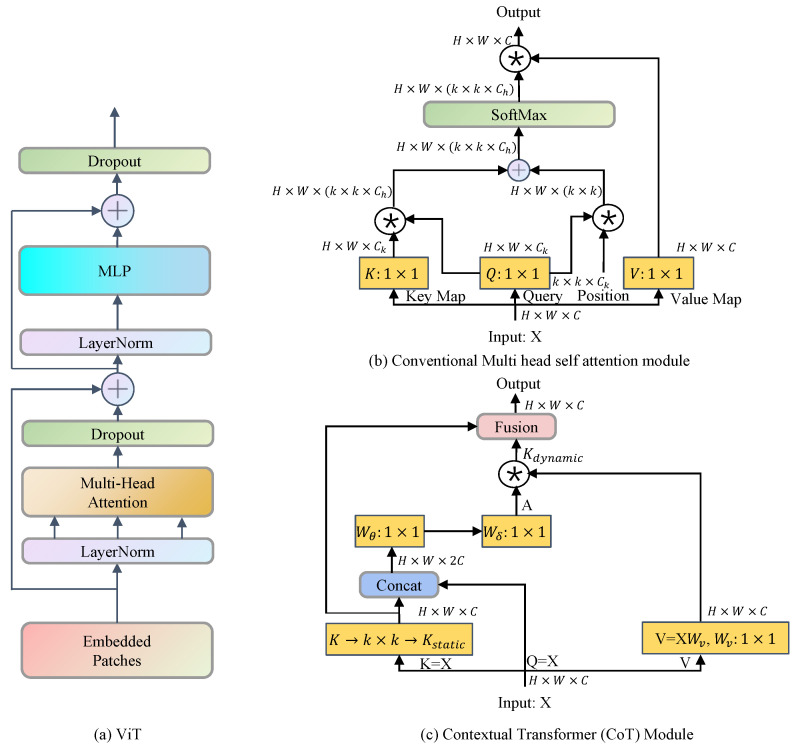
A variety of transformer stylized self-attention modules.

The traditional multi-head self-attention mechanism widely adopted in the visual model backbone in transformer-based approaches [52], as shown in Figure 2b, is capable of triggering feature interactions between different spatial locations. However, this mechanism has limited capacity to perform visual representation learning on 2D feature maps, as it does not explore the rich contextual information between query–key pairs, which are learned independently through isolated query–key pairs. To address this issue, Li et al. [15] proposed a new transformer-based module, the contextual transformer (CoT) block (Figure 2c), which integrated contextual information mining and self-attention learning into a unified architecture.

Given an input 2D feature map *X* of size H×W×C (*C*: channel, *H*: height, *W*: width), the keys, queries, and values were obtained according to Q=X, K=X, and $V=XW_{v}$. $W_{v}$ means the embedding matrix which is implemented as a 1×1 convolution in space. As opposed to a traditional self-attention mechanism, the CoT module first applied a group convolution of k×k to extract contextual information. The obtained $K_{static}$ reflected the contextual information between adjacent key values, which was referred to as static contextual representation. Subsequently, after concatenating $K_{static}$ and *Q*, the following attention matrix *A* was obtained through two consecutive 1×1 convolutional operations, where $W_{\theta }$ indicates that the ReLU activation function was used, whereas $W_{\delta }$ does not. The [] in Equation (2) indicate concatenation, which is accomplished by joining two matrices along a certain dimension. Just as in Figure 2c, after the concat module, H×W×C becomes H×W×2C.

(2) $$A=\left[K_{static},Q\right]W_{\theta }W_{\delta }$$

At each position, the local correlation matrix was learned from the queries and keys, rather than the independent query–key pairs, which enhanced the learning capacity of the self-attention mechanism by exploiting the static contextual information, thus leading to feature-mapping (⊛ represents the local matrix multiplication operation):

(3) $$K_{dynamic}=V⊛A$$

where $K_{dynamic}$ is a dynamic context representation, capturing feature interactions between inputs. The output of the CoT block was a fusion of static ($K_{static}$) and dynamic contexts ($K_{dynamic}$) by an attention mechanism. As compared to traditional multi-head self-attention modules, the CoT block was able to fully implement the input contextual information to guide the training of the dynamic attention matrix, thereby enhancing its visual expression ability. Additionally, the CoT block was a plug-and-play module, which enabled the direct replacement of convolutional modules in existing neural network models. In this paper, three CoT blocks were used to construct the CoT3 in Layers 2, 4, 6, and 9 (see Table 2). This combination of convolutional and CoT3 layers enabled the model to benefit from the local feature extraction of the convolutional modules and the contextual information capture of transformer modules, thus enabling better integration of the local and global information.

#### 3.4.2. Attention Block

The transformer’s self-attention mechanism has had considerable success in the field of computer vision due to its ability to capture internal correlations in the data and features without relying on external information. However, attention mechanisms are not limited to self-attention, and this section explores the potential of introducing other attention mechanisms into the model in order to further improve its capacity to capture global information.

Existing attention modules in computer vision have largely focused on the channel and spatial domains, which are analogous to the feature- and spatial-based attention in human brains. Channel attention is a one-dimensional approach, whereby each channel is treated differently while all positions are treated equally. Spatial attention, on the other hand, is two-dimensional, with each position being treated differently while all channels are treated equally. Several studies, such as BAM [54] and CBAM [55], have proposed parallel or serialized approaches to spatial and channel attention. However, in human brains, these two attention aspects often work collaboratively. To address this, Yang et al. [16] proposed a unified weight-attention module, a simple attention module (SimAM), to perform operations similar to those of human brains, enabling each neuron to be assigned a unique weight. SimAM defined the energy function of each neuron as follows:

(4) $$e_{t}\left(w_{t},b_{t},y,x_{i}\right)={\left(y_{t}-\hat{t}\right)}^{2}+\frac{1}{M-1}\sum_{i=1}^{M-1}{\left(y_{o}-{\hat{x}}_{i}\right)}^{2}$$

The linear transformations of the target neuron and other neurons in the single channel of the input feature $X\in R^{C\times H\times W}$ (*C*: channel, *H*: height, *W*: width) are, respectively, denoted as $\hat{t}=w_{t}t+b_{t}$ and ${\hat{x}}_{i}=w_{t}x_{i}+b_{t}$, where *t* and $x_{i}$ are the indices in the spatial dimension and M=H×W represents the number of neurons in the channel. The variables $w_{t}$ and $b_{t}$ are the weights and biases of the linear transformation, respectively. In Equation (4), the minimum value was achieved when the value of $\hat{t}$ was equal to $y_{t}$, and all other values of ${\hat{x}}_{i}$ were equal to $y_{o}$, where $y_{t}$ and $y_{o}$ are two distinct values.

The existing attention modules that operate within the channel and spatial dimensions suffer from two significant limitations: firstly, they refine features only in one dimension of a channel or space; and secondly, their structures often necessitate complex operations, such as pooling. In contrast, SimAM represented a conceptually simple yet highly effective attention mechanism for CNNs. Specifically, SimAM assigned a three-dimensional attention weight that did not increase the number of parameters needed. To evaluate its efficacy, we introduced a SimAM layer after the 23rd layer in our detector, as indicated in Table 3. Through this approach, our model could capture comprehensive global contextual information after the convolutional layer and COT3 layer were connected.

By integrating CNN layers with CoT3 layers and a SimAM layer, we developed the GL-YOLO model, which enabled the integration of global contextual information with local information for improved object detection. To achieve this, we first obtained the proposed GL-YOLO architecture via the previously mentioned research. Additionally, we utilized *K*-means++ to cluster the dataset and generate suitable anchors, resulting in further enhancements to the model’s detection performance. Ultimately, the combination of these design elements contributed to the superior performance of the GL-YOLO model in object-detection tasks.

### 3.5. Lightweight Model Structure Design

The combination of the transformer and attention modules with GL-YOLO has been demonstrated to substantially enhance the detection performance of the model. However, the detection speed of the model was also a crucial factor. This section describes the methods considered to optimize the model’s structure in order to achieve a better balance between accuracy and detection speed.

#### 3.5.1. Stem Block

Recent studies have shown that the focus module utilized in YOLOv5 [17] was not optimal in terms of efficiency and implementation in most deep learning frameworks [11]. To address this, we opted to replace it with a stem module [18] entirely composed of standard convolutional units. Prior to discussing the parameters and FLOPs associated with both the standard convolutional and focus operations, we provide the following formulas for their calculations. Specifically, consider a convolutional layer with dimensions of h×w×c×n, where *h* and *w* represent the height and width, respectively, while *c* and *n* denote the input and output channels, respectively.

(5) No.ofParameters=n×(h×w×c+1)

(6) FLOPs=H×W×n×(h×w×c+1)

where *H* and *W* represent the height and width of the resulting feature map, respectively, measured in pixels. To determine the number of parameters and operations required for both the convolutional operation and the focus module, we employed a single image with dimensions of 640 × 640 × 3.

- Convolution. The convolutional operation employed a 3 × 3 kernel, a stride of 2, and an output channel of 32, resulting in a feature map of 320 × 320 × 32 after down-sampling.
  (7) $$\begin{array}{cc} \mathrm{No}.\;\mathrm{of}\;\mathrm{Parameters}\;(\mathrm{Convolution}) & =32\times (3\times 3\times 3+1)\\ \; & =3\times 3\times 3\times 32+32\\ \; & =896 \end{array}$$
  (8) $$\begin{array}{cc} \mathrm{FLOPs}\;(\mathrm{Convolution}) & =320\times 320\times 32\times (3\times 3\times 3+1)\\ \; & =3\times 3\times 3\times 320\times 320\times 32+320\times 320\times 32\\ \; & =91,750,400 \end{array}$$
- Focus. The focus module operated on an input image by slicing it before it entered the backbone of the network. Specifically, this involved selecting a value for every other pixel in the image, similar to the nearest-neighbor down-sampling method, resulting in four images that retained all original information. Through this approach, *W* and *H* could be concentrated in the channel space, expanding the input channels by a factor of 4, or 12 channels when using an RGB 3-channel image. Ultimately, the newly obtained image was subjected to a convolutional operation, yielding a feature map that was twice the down-sampling result without any loss of information. By inputting a 640 × 640 × 3 image into the focus module and applying the slicing operation, the image was first transformed into a 320 × 320 × 12 feature map, which then underwent additional convolutional operations and, ultimately, resulted in a 320 × 320 × 32 feature map. By utilizing these steps, we obtained the parameters and FLOPs associated with the focus module, as follows:
  (9) $$\begin{array}{cc} \mathrm{No}.\;\mathrm{of}\;\mathrm{Parameters}\;(\mathrm{Focus}) & =32\times (3\times 3\times 12+1)\\ \; & =3\times 3\times 12\times 32+32\\ \; & =3488 \end{array}$$
  (10) $$\begin{array}{cc} \mathrm{FLOPs}\;(\mathrm{Focus}) & =320\times 320\times 32\times (3\times 3\times 12+1)\\ \; & =3\times 3\times 12\times 320\times 320\times 32+320\times 320\times 32\\ \; & =357,171,200 \end{array}$$

**Figure 3:**
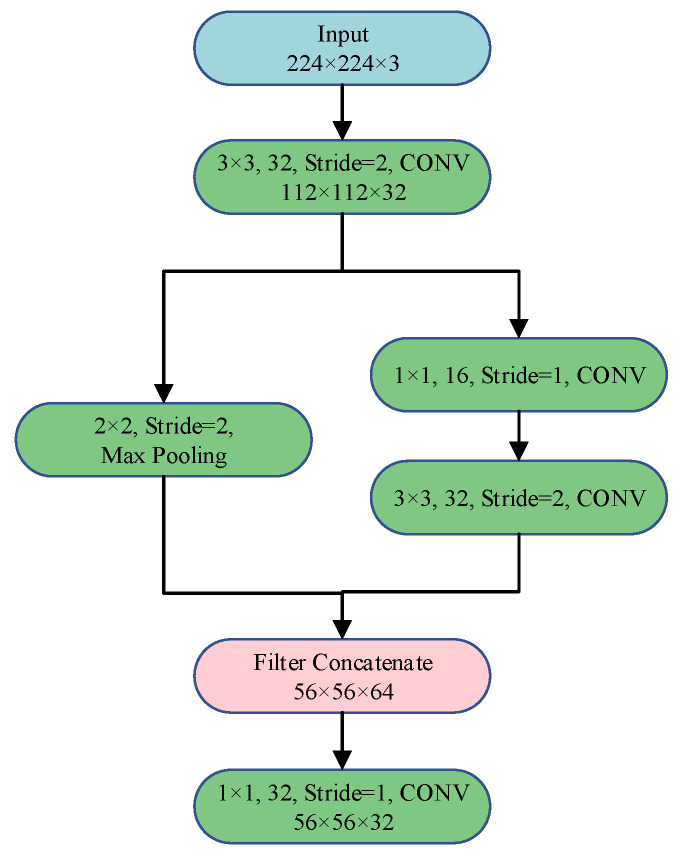
Stem module’s structure.

A comparison between the focus module and single-layer convolution revealed that the former had about 400% more parameters and FLOPs. Additionally, while the standard convolution could be readily adapted to various formats, such as ONNX, TensorFlow, and TensorFlow Lite, the same could not be said for the focus module, which was not a generic structure and was not widely supported by many deep-learning frameworks. Given the factors of parameter count, FLOPs, and model applicability, this study proposed replacing the focus module with a stem module [18]. As opposed to the focus module, the stem module provided a plug-and-play solution that offered richer feature expression without incurring additional computational overhead. Figure 3 shows the specific structure of the stem module, which consisted of a 3 × 3 convolution with a stride of 2 for rapid reductions in dimensionality, followed by a dual-branch structure, with one branch using a 3 × 3 convolution with a stride of 2, and the other branch using a max-pooling layer.

#### 3.5.2. Rep Block

Recent developments in computer vision have resulted in the emergence of deep learning models that outperform traditional CNNs by utilizing complex structural designs [48,56]. However, such models often include limitations, including challenges related to implementation and customization due to their multi-branch design incurring slower inference speeds and reduced memory utilization. Additionally, some model components, such as the depth convolution in Xception [57], the channel shuffle operation in ShuffleNet [58], and the depthwise separable convolution in MobileNets [46], have increased memory access costs and lack support for various devices. To overcome these challenges, networks such as ACNet [59] and RepVGG [19] have been proposed, both of which employ a technique known as structural re-parameterization. This enables a multi-branch structure during training and a single-path model during deployment and inference, thereby combining the high performance of multi-branch structures with the speed of single-path models. Building upon prior research [11,19], we introduced rep modules with structural re-parameterization capabilities into GL-YOLO (see Table 4). This enabled us to decouple training and inference via structural re-parameterization, leveraging a multi-branch structure to enhance performance during training while re-parameterizing to a single 3 × 3 convolutional structure to accelerate inference.

#### 3.5.3. Lightweight Detection Head

To reduce the complexity of our model, we simplified the neck and head components, as depicted in Table 5. Through this modification, we removed a considerable number of channels, leading to a substantial reduction in the computational cost of the model.

In summary, we have presented GL-YOLO-Lite, the final model proposed in this paper. GL-YOLO-Lite incorporated the stem module as a replacement for the original focus module, as well as the rep modules with re-parameterization technology to optimize the neck and head components of the model. Through these modifications, we achieved a notable reduction in parameters and FLOPs while maintaining an excellent balance between detection accuracy and inference speed.

## 4. Datasets

### 4.1. Fallen Person Detection Dataset

In the field of FPD, having access to a reliable dataset is crucial for improving the performance of detection modeling. However, collecting fallen person images in real-world scenarios presents significant difficulties, and most existing public datasets for FPD have been captured in simple experimental environments that did not accurately reflect the complexity of real-life scenarios. Therefore, it was necessary to construct an FPD dataset that was representative of real-world scenarios to meet research needs. This study used two methods to obtain the required images: (1) the conversion of videos containing fall scenes taken by surveillance systems into images; and (2) the use of web-crawler technology to obtain online images of human falls in real-life scenarios. The authors obtained a total of 4569 images through these two methods, and then they utilized an open-source tool, LabelImg [60], to uniformly label the dataset images and generate the corresponding labels. The label set for the FPD was “fall”, with a total of 4576 objects labeled as such in the dataset. Finally, the authors divided the FPD dataset (FPDD) into training, testing, and validation sets using an 80/16/4 ratio.

### 4.2. PASCAL VOC Dataset

**Figure 4:**
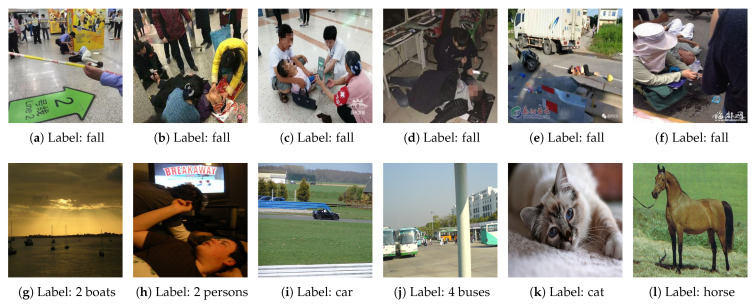
Examples of the FPDD and Pascal VOC datasets, (**a**–**f**) and (**g**–**l**), respectively. Please note that we resized these images so that they could be better displayed.

Pascal VOC [20,21] is a widely employed benchmark dataset for visual target classification, recognition, and detection tasks. It comprised two versions: VOC2007 and VOC2012. The former consisted of 9963 annotated images with a total of 24,640 objects annotated, which were divided into training, testing, and validation sets. VOC2012 was an upgraded version of VOC2007 containing 11,530 images and 27,450 objects in the training, testing, and validation sets. Notably, VOC2012 was mutually exclusive of VOC2007. To train our model, we utilized the commonly applied 07+12 method [10,11], which employed the VOC2007 training and validation sets, as well as the VOC2012 training and validation sets for training; and the VOC2007 testing set for testing. Figure 4 displays samples from both the FPDD and Pascal VOC datasets.

## 5. Experiments

### 5.1. Metrics and Implementation

#### 5.1.1. Metrics

In the field of object detection, mAP and FPS are widely accepted metrics for assessing the accuracy and speed, respectively, of detection algorithms.In addition to these metrics, practical applications of the algorithms studied in this paper required the inclusion of evaluation indicators such as parameters and giga-FLOPs (GFLOPs). To comprehensively evaluate the proposed model, the authors employed the technique for order of preference by the similarity to ideal solution (TOPSIS) method, whereby the four indicators were weighted as follows: mAP, 40%; FPS, 20%; parameters, 20%; and GFLOPs, 20%. Among these, mAP and FPS are extremely large indicators, while parameters and GFLOPs are extremely small indicators. TOPSIS is a commonly used comprehensive evaluation approach that fully leverages the original data information, generating results that accurately reflect the discrepancies between various evaluation schemes.

#### 5.1.2. Implementation

This study was conducted on a workstation equipped with an Intel E5-2620 v4 @ 2.10 GHz CPU, an NVIDIA Titan XP (12 GB) GPU, and 16 GB RAM. To regenerate new anchors for the FPDD dataset, the *K*-means++ clustering algorithm was applied, which yielded anchor sizes of (137 × 119), (190 × 224), (223 × 377), (304 × 155), (331 × 288), (359 × 459), (459 × 219), (513 × 358), and (549 × 545). For the Pascal VOC dataset, the new anchors were (48 × 78), (73 × 204), (134 × 336), (142 × 126), (224 × 450), (256 × 223), (361 × 505), (480 × 298), and (567 × 545). The detailed configurations of the training and testing environments, as well as the hyperparameter settings for GL-YOLO-Lite, are provided in Table 6.

### 5.2. Comparison with the State-of-the-Art Modeling

This study compared the performance of the proposed GL-YOLO-Lite to that of other state-of-the-art lightweight object-detection models, including MobileNetV3 [46], ShuffleNetV2 [58], and GhostNet [61], on the FPDD. Furthermore, to assess the generalization capacity of GL-YOLO-Lite, comparison experiments were conducted on the more challenging and publicly available Pascal VOC dataset. Table 7 and Table 8 present the comparison results among GL-YOLO-Lite and seven other advanced lightweight object-detection methods on the FPDD and Pascal VOC datasets, respectively. The best performance is in bold font for a particular index, while the second-best performance is underlined. The results from Table 7 and Table 8 revealed the following:

- The proposed GL-YOLO series algorithms exhibited superior model detection accuracy, as determined by mAP@0.5, on both the FPDD and the Pascal VOC datasets. On the FPDD, GL-YOLO achieved the highest mAP@0.5 of 89.1%, while GL-YOLO-Lite achieved 88.5%. Even though YOLOv5-s attained the highest mAP@0.5 of 85.7%, among other advanced object-detection models, it still lagged behind GL-YOLO-Lite by 2.8%. Similarly, on the Pascal VOC dataset, GL-YOLO again achieved the highest mAP@0.5 of 82.5%, while GL-YOLO-Lite attained an mAP@0.5 of 80%. The highest mAP@0.5 of 78.2% was obtained by YOLOv5-Lite-g [62] on this dataset, but this result still fell short of GL-YOLO-Lite by 1.8%. These findings suggested that the transformer and attention modules in GL-YOLO-Lite effectively enhanced the feature extraction capability of the model by fully utilizing global contextual information and, thereby, improving its object-detection performance.
- While GL-YOLO achieved the highest mAP@0.5 values on both datasets, its sub-optimal speed performance was limited by its model structure. However, GL-YOLO-Lite (FPDD: 88.5% mAP@52.63 FPS, Pascal VOC: 80% mAP@56.82 FPS) ranked high in terms of mAP (second in the FPDD and the Pascal VOC dataset) and was in the middle tier in terms of FPS. As compared to the baseline model YOLOv5-s, GL-YOLO-Lite was a significant improvement in terms of the parameters GFLOPs, mAP@0.5, and FPS-CPU, with only a slight reduction in FPS-GPU, indicating the effectiveness of the proposed algorithms presented in this paper.
- The comprehensive rankings in the last column of Table 7 and Table 8 illustrated that GL-YOLO-Lite outperformed the baseline YOLOv5-s model, achieving the highest TOPSIS scores (FPDD: 0.573961, Pascal VOC: 0.563583). These results demonstrated that GL-YOLO-Lite was a significant advancement, as compared to YOLOv5-s, due to its robust feature extraction and efficient structural design with significantly fewer parameters and GFLOPs, while still maintaining a high object-detection precision (mAP). Furthermore, its real-time processing speed (FPS greater than 30 FPS) on the desktop GPU Titan Xp indicated its potential for handling FPD on typical workstations.

In FPD, precision, recall, and F1 score serve as commonly employed metrics to evaluate model performance. Precision, a vital metric that concerns predicted outcomes, quantifies the likelihood of true-positive samples among all samples forecasted as positive. Its mathematical expression is formulated as follows:

(11) $$\mathrm{Precision}=\frac{\mathrm{TP}}{\mathrm{TP}+\mathrm{FP}}$$

where true positive (TP) signifies the correct classification of positive examples as positive, whereas false positive (FP) denotes the incorrect labeling of negative samples as positive.

Recall pertains to the original sample and signifies the likelihood of positive samples being predicted as positive. The mathematical expression for this probability was determined as follows:

(12) $$\mathrm{Recall}=\frac{\mathrm{TP}}{\mathrm{TP}+\mathrm{FN}}$$

where false negative (FN) denotes the false labeling of positive examples as negative.

The F1 score is a metric that considers both precision and recall, with the aim of achieving an equilibrium between the two factors while maximizing their values. The expression for calculating the F1 score was the following:

(13) $$F1\;\mathrm{score}=\frac{2\times \mathrm{Precision}\times \mathrm{Recall}}{\mathrm{Precision}+\mathrm{Recall}}$$

Table 9 presents a detailed assessment of the precision, recall, mAP@0.5, and F1 score of GL-YOLO-Lite and YOLOv5s, offering a comprehensive evaluation of their performance. GL-YOLO-Lite demonstrated a significant improvement in both its F1 score (i.e., an increase of 0.026) and mAP@0.5 (i.e., an increase of 0.028) relative to YOLOv5s, providing additional evidence of its exceptional performance.

### 5.3. Ablation Study and Visualization

In this study, we analyzed the effectiveness of various components by incorporating them into a baseline model YOLOv5s, which attained a mAP@0.5 of 77.7% on the Pascal VOC dataset. The examined components included newly generated anchors using *K*-means++, a lightweight detection head, as well as transformer, attention, stem, and rep modules. Table 10 displays the performance of different staged models. Our findings indicated that the mAP@0.5 was enhanced from 77.7% to 82.5% when the new anchors had been generated using *K*-means++ with the transformer and attention modules simultaneously integrated, resulting in a GL-YOLO model with 7.07 parameters and 16.4 GFLOPs. Building upon the GL-YOLO, we further appended the stem and rep modules, along with the use of a lightweight detection head, to obtain the GL-YOLO-Lite model. This model achieved a mAP@0.5 of 80%, with 4.42 parameters and 3.4 GFLOPs. As compared to the baseline YOLOv5s model, GL-YOLO-Lite demonstrated a reduction of 37.83% in parameters and 79.27% in GFLOPs. The outcomes of our experiments underscored the significant contributions of each individual component in the GL-YOLO-Lite architecture.

**Figure 5:**
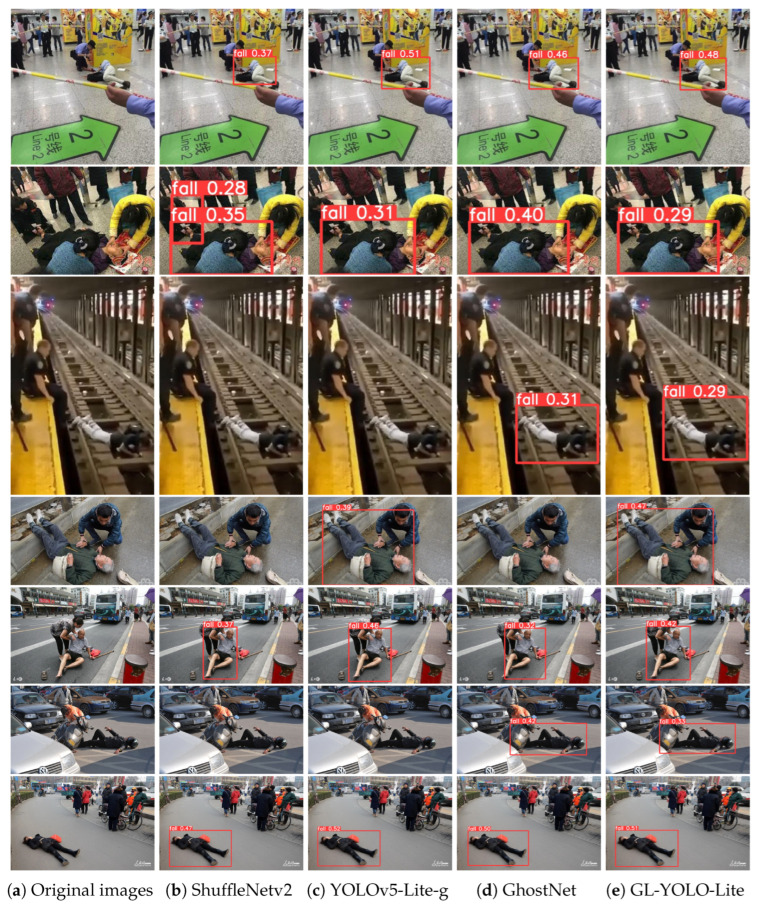
Detection results by GL-YOLO-Lite and other representative algorithms. The ground truth for each image was fall. The images in the Rows 3–7 were collected from the web separately and were not included in the FPDD.

Figure 5 displays the partial visualized results of the comparison of GL-YOLO-Lite to other advanced lightweight detection models on the FPDD. Our study emphasized the effectiveness of GL-YOLO-Lite in accurately detecting the targets in the images, which was achieved by incorporating global contextual information with local features. This combination led to a significant improvement in detection accuracy. Furthermore, in contrast to the other models, GL-YOLO-Lite demonstrated superior robustness in detection accuracy even when utilizing images beyond those in the FPDD (Rows 3–7). This observation highlighted the model’s robustness and exceptional generalization capability.

### 5.4. Experiments on a Mobile Phone

**Figure 6:**
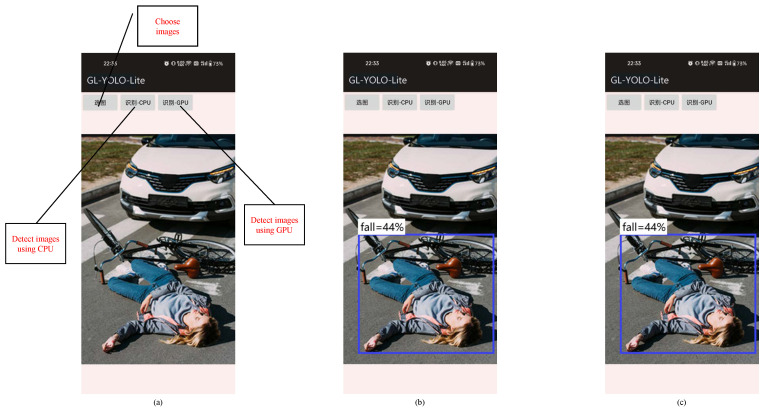
The Android application: (**a**) the app user interface, (**b**) images detected by the CPU, and (**c**) images detected by the GPU.

This study also presented a comprehensive evaluation of the deployability of the proposed GL-YOLO-Lite algorithm. To conduct this evaluation, we deployed various lightweight algorithms on an Honor V20 device utilizing the NCNN [63] framework and compared their actual detection speeds. As demonstrated in Table 11, the computational capacity of the Honor V20 device was not particularly robust. To execute the assessments, we developed a detection application, as shown in Figure 6, that supported the loading of different model weights onto the application. The algorithm’s weight was loaded onto the application, and both the CPU and GPU of the device were utilized to perform 15 detection operations per image. Subsequently, the duration for each detection was recorded, and the average of the 15 detection times was computed to determine the final time requirements for the algorithm’s detection. The results of this analysis are presented in Table 12. As shown in Table 12, as compared to other lightweight detection models, GL-YOLO-Lite achieved the quickest detection speed (60.80 ms). After analyzing Table 7, Table 8 and Table 12, our findings indicated the GL-YOLO-Lite proposed in this paper achieved an improved compromise between FPS and mAP relative to other advanced lightweight models, regardless of the platform utilized (desktop GPU, workstation CPU, or mobile platform).

## 6. Conclusions

This work presented a novel model, GL-YOLO-Lite, specifically designed for FPD to address the limitations of existing deep-learning-based object-detection algorithms. These algorithms have been restricted to utilizing information solely from within the candidate object region and lack the ability to capture global information, which has limited their detection accuracy while also having considerably high computational costs. In contrast, the integration of transformer and attention modules into our model enabled the effective learning and fusion of global–local feature information, resulting in improved detection accuracy and generalization capability. The GL-YOLO-Lite architecture achieved reductions in parameters and FLOPs by excluding the initial focus module, adopting stem and rep modules, and employing a novel detection head. Although this compromised detection accuracy slightly, it significantly improved the detection speed, achieving an excellent balance between speed and accuracy. To evaluate the performance and efficiency of GL-YOLO-Lite, we constructed the FPDD of various real-world scenarios of human falls. The results of numerous experiments demonstrated the remarkable performance and efficiency of GL-YOLO-Lite, as it achieved good performance on the FPDD and the PASCAL VOC dataset with relatively low computational overhead. Using mAP@0.5, FPS, FLOPs, and parameters as the evaluation indicators, as well as TOPSIS as the comprehensive evaluation method, our model obtained the highest TOPSIS score, fully demonstrating the excellence of GL-YOLO-Lite.

## Figures and Tables

**Table 1:** Comparison of various methods of FPD technologies.

FPD Technology	Main Technical Principles	Highlights	Limitations
Scene perception	Infrared sensors,	Non-intrusiveness	The device has a single deployment environment
	radar technology,	Real-time performance	High false alarm rate;
	millimeter wave radar, etc.	Scalability	The device is expensive
Wearable device	Accelerometers	Easy to use	Long-term wear reduces comfort
	Gyroscopes	Strong applicability	Battery life issues
	Magnetometers	Lower cost	High hardware and software requirements
Visual information	Camera captures data	Non-intrusiveness	Poor quality of anchor box generation
	Machine learning	Easy installation	Inadequate utilization of global features
	Deep learning	Visual effectiveness	Huge parameters and FLOPs
GL-YOLO-Lite	Automatically generating high-quality anchors		
	Combining global contextual information and local features using transformer and attention modules to improve model detection accuracy, robustness		
	Reducing parameters and FLOPs while increasing detection speed using stem module, rep modules, and redesigned lightweight detection head		

**Table 2:** Backbones of YOLOv5 and GL-YOLO.

	YOLOv5				GL-YOLO			
	**Input**	**Module**	**Number of Modules**	**Args**	**Input**	**Module**	**Number of Modules**	**Args**
Layer 0	Image	Focus	1	(64, 3)	Image	Focus	1	(64, 3)
Layer 1	Layer 0	Conv	1	(128, 3, 2)	Layer 0	Conv	1	(128, 3, 2)
Layer 2	Layer 1	C3	3	(128)	Layer 1	**CoT3**	3	(128)
Layer 3	Layer 2	Conv	1	(256, 3, 2)	Layer 2	Conv	1	(256, 3, 2)
Layer 4	Layer 3	C3	9	(256)	Layer 3	**CoT3**	9	(256)
Layer 5	Layer 4	Conv	1	(512, 3, 2)	Layer 4	Conv	1	(512, 3, 2)
Layer 6	Layer 5	C3	9	(512)	Layer 5	**CoT3**	9	(512)
Layer 7	Layer 6	Conv	1	(1024, 3, 2)	Layer 6	Conv	1	(1024, 3, 2)
Layer 8	Layer 7	SPP	1	(1024, (5, 9, 13))	Layer 7	SPP	1	(1024, (5, 9, 13))
Layer 9	Layer 8	C3	3	(1024, False)	Layer 8	**CoT3**	3	(1024, False)

**Table 3:** Head in YOLOv5 and GL-YOLO.

	YOLOv5				GL-YOLO			
	**Input**	**Module**	**Number of Modules**	**Args**	**Input**	**Module**	**Number of Modules**	**Args**
Layer 21	Layer 20	Conv	1	(512, 3, 2)	Layer 20	Conv	1	(512, 3, 2)
Layer 22	Layer 21 + Layer 10	Concat	1	(1)	Layer 21 + Layer 10	Concat	1	(1)
Layer 23	Layer 22	C3	3	(1024, False)	Layer 22	C3	3	(1024, False)
Layer 24	-	-	-	-	Layer 24	**SimAM**	1	(1024)

**Table 4:** Backbones in GL-YOLO and GL-YOLO-Lite.

	GL-YOLO				GL-YOLO-Lite			
	**Input**	**Module**	**Number of Modules**	**Args**	**Input**	**Module**	**Number of Modules**	**Args**
Layer 0	Image	Focus	1	(64, 3)	Image	**Stem block**	1	(64, 3)
Layer 1	Layer 0	Conv	1	(128, 3, 2)	Layer 0	**Rep block**	1	(128, 3, 2)
Layer 2	Layer 1	CoT3	3	(128)	Layer 1	CoT3	3	(128)
Layer 3	Layer 2	Conv	1	(256, 3, 2)	Layer 2	**Rep block**	1	(256, 3, 2)
Layer 4	Layer 3	CoT3	9	(256)	Layer 3	CoT3	9	(256)
Layer 5	Layer 4	Conv	1	(512, 3, 2)	Layer 4	**Rep block**	1	(512, 3, 2)
Layer 6	Layer 5	CoT3	9	(512)	Layer 5	CoT3	9	(512)
Layer 7	Layer 6	Conv	1	(1024, 3, 2)	Layer 6	**Rep block**	1	(1024, 3, 2)
Layer 8	Layer 7	SPP	1	(1024, (5, 9, 13))	Layer 7	SPP	1	(1024, (5, 9, 13))
Layer 9	Layer 8	CoT3	3	(1024, False)	Layer 8	CoT3	3	(1024, False)

**Table 5:** Heads in GL-YOLO and GL-YOLO-Lite.

	GL-YOLO				GL-YOLO-Lite			
	**Input**	**Module**	**Number of Modules**	**Args**	**Input**	**Module**	**Number of Modules**	**Args**
Layer 10	Layer 9	Conv	1	(512, 1, 1)	Layer 9	Conv	1	(**128**, 1, 1)
Layer 11	Layer 10	Up-Sample	1	(None, 2, ’nearest’)	Layer 10	Up-Sample	1	(None, 2, ’nearest’)
Layer 12	Layer 6 + Layer 11	Concat	1	(1)	Layer 6 + Layer 11	Concat	1	(1)
Layer 13	Layer 12	C3	3	(512, False)	Layer 12	C3	3	(**128**, False)
Layer 14	Layer 13	Conv	1	(256, 1, 1)	Layer 13	Conv	1	(**128**, 1, 1)
Layer 15	Layer 14	Up-Sample	1	(None, 2, ’nearest’)	Layer 14	Up-Sample	1	(None, 2, ’nearest’)
Layer 16	Layer 4 + Layer 15	Concat	1	(1)	Layer 4 + Layer 15	Concat	1	(1)
Layer 17	Layer 16	C3	3	(256, False)	Layer 16	C3	3	(**128**, False)
Layer 18	Layer 17	Conv	1	(256, 3, 2)	Layer 17	Conv	1	(**128**, 3, 2)
Layer 19	Layer 14 + Layer 18	Concat	1	(1)	Layer 14 + Layer 18	Concat	1	(1)
Layer 20	Layer 19	C3	3	(512, False)	Layer 19	C3	3	(**128**, False)
Layer 21	Layer 20	Conv	1	(512, 3, 2)	Layer 20	Conv	1	(**128**, 3, 2)
Layer 22	Layer 10 + Layer 21	Concat	1	(1)	Layer 10 + Layer 21	Concat	1	(1)
Layer 23	Layer 22	C3	3	(1024, False)	Layer 22	C3	3	(**128**, False)
Layer 24	Layer 23	SimAM	1	(1024)	Layer 23	SimAM	1	(**128**)

**Table 6:** Workstation configuration and the hyperparameters of GL-YOLO-Lite.

Workstation		Hyperparameters of GL-YOLO-Lite	
CPU	Intel(R) Xeon(R) CPU E5-2620 v4 @ 2.10 GHz	Initial Learning Rate	0.01
GPU	TITAN Xp(12 GB)	Optimizer	Adam
Memory	16 GB	Momentum	0.937
Operating System	ubuntu18.04	Weight Decay	0.0005
Deep Learning Framework	PyTorch 1.7.0	IoU Threshold	0.45
CUDA version	11	Training Epochs	300

**Table 7:** Results on FPDD. The best results are presented in bold, and the second-best results are underlined.

Methods		Backbone	Input Size	Parameters	GFLOPs	mAP@0.5(%)	FPS-GPU	FPS-CPU	TOPSIS Score	Ranking
1	YOLOv5-mbv3-small	MobileNetv3-small [46], ICCV	640 × 640	3.54	6.3	80.1	55.25	13.40	0.458764	6
2	YOLOv5-mbv3-large	MobileNetv3-large [46], ICCV		5.2	10.3	83.9	47.62	7.50	0.485195	3
3	YOLOv5-ShuffleNetv2	ShuffleNetv2 [58], ECCV		**0.44**	**1.3**	70.9	52.91	**18.32**	0.457568	7
4	YOLOv3-Tiny	Darknet-53 [43]		8.67	12.9	69.6	**243.90**	6.83	0.457558	8
5	YOLOv5-s	CSPDarknet-SPP [17]		7.05	16.3	85.7	73.53	7.03	0.440520	9
6	YOLOv5-lite-g	RepVGG [19], CVPR		5.3	15.1	85.5	62.89	6.98	0.468680	5
7	YOLOv5s-Ghost	GhostNet [61], CVPR		3.68	8.1	86.1	56.18	7.71	0.555261	2
8	GL-YOLO	GL-YOLO	640 × 640	7.03	16.2	**89.1**	49.75	5.51	0.469409	4
9	GL-YOLO-Lite	GL-YOLO		4.41	3.3	88.5	52.63	9.89	**0.573961**	**1**

**Table 8:** Results on PASCAL VOC dataset. The best results are presented in bold, and the second-best results are underlined.

Methods		Backbone	Input Size	Parameters	GFLOPs	mAP@0.5(%)	FPS-GPU	FPS-CPU	TOPSIS Score	Ranking
1	YOLOv5-mbv3-small	MobileNetv3-small [46], ICCV	640 × 640	3.59	6.4	69.1	61.35	15.11	0.438815	8
2	YOLOv5-mbv3-large	MobileNetv3-large [46], ICCV		5.25	10.3	77	54.95	8.01	0.500519	3
3	YOLOv5-ShuffleNetv2	ShuffleNetv2 [58], ECCV		**0.45**	**1.4**	56.7	61.35	**19.19**	0.453154	7
4	YOLOv3-Tiny	Darknet-53 [43]		8.71	13	57.7	**277.78**	7.58	0.462992	6
5	YOLOv5-s	CSPDarknet-SPP [17]		7.11	16.4	77.8	69.93	8.08	0.431950	9
6	YOLOv5-lite-g	RepVGG [19], CVPR		5.32	15.3	78.2	70.42	7.86	0.471702	4
7	YOLOv5s-Ghost	GhostNet [61], CVPR		3.73	8.3	77	61.73	8.29	0.549522	2
8	GL-YOLO	GL-YOLO	640 × 640	7.08	16.4	**82.5**	51.55	6.43	0.467550	5
9	GL-YOLO-Lite	GL-YOLO		4.42	3.4	80	56.82	11.07	**0.563583**	**1**

**Table 9:** Comparison of additional metrics between GL-YOLO-lite and YOLOv5s.

Methods	Precision	Recall	F1 Score	mAP@0.5
YOLOv5-s	0.812	0.838	0.825	0.857
GL-YOLO-Lite	0.843	0.859	0.851	0.885

**Table 10:** Ablation study of GL-YOLO-Lite on the PASCAL VOC dataset.

Methods		Components						Input Size	Parameters	GFLOPs	mAP@0.5(%)
1	YOLOv5s							640 × 640	7.11	16.4	77.7
2	*K*-means++	✓						640 × 640	7.11	16.4	79.5
3	Transformer block	✓	✓						7.08	16.4	82
4	Attention block	✓	✓	✓					7.07	16.4	82.5
5	Stem block	✓	✓	✓	✓				7.09	4.5	80.9
6	Rep block	✓	✓	✓	✓	✓			7.09	4.5	80.3
7	Lighter head	✓	✓	✓	✓	✓	✓		4.42	3.4	80

**Table 11:** Configuration of Honor V20.

Configuration of Honor V20	
Brand	Honor
Model	V20
System on Chip	HiSilicon Kirin 980
CPU	2 × A76 2.6 GHz + 2 × A76 1.92 GHz + 4 × A55 1.8 GHz
GPU	Mali-G76 MP10 (720 MHz): 691 GFLOPs
Random Access Memory	8 GB
Operating System	Android 11

**Table 12:** Comparison of the detection speed of GL-YOLO-Lite and other algorithms on Honor V20. The best results are presented in bold, and the second-best results are underlined.

Methods	Honor V20	Time (ms)															Trimmed Mean (ms)
YOLOv5s	CPU	116.24	104.46	113.28	102.41	107.4	122.67	96.46	96.81	105.21	106.73	123.78	97.17	104.6	84.76	95.3	105.29
	GPU	200.13	130.42	192.44	188.62	205.3	125.33	193.83	195.48	189.53	182	200.83	130.57	193.9	128.19	130.06	173.54
YOLOv5s-Ghost	CPU	104.46	87.67	75.2	75.62	68.19	79.98	67.59	82.66	71.47	73.07	71.9	78.42	80.02	89.8	69.55	77.20
	GPU	258.94	79.87	72.82	92.29	75	93.2	74.6	125.18	75.82	95.62	76.5	109.59	75.37	93.58	74.84	87.80
GL-YOLO	CPU	61.78	117.57	48.19	57.03	59.07	56.84	48.55	62.62	49.53	64.03	52.03	64.90	49.12	59.97	108.68	63.99
	GPU	69.58	67.91	104.79	75.90	64.19	100.88	104.79	74.05	106.31	105.62	83.42	78.41	107.60	76.49	89.47	87.29
GL-YOLO-Lite	CPU	76.75	41.56	46.27	48.02	52.74	70.15	68.94	59.48	73.90	75.24	72.70	44.00	44.27	71.11	66.88	**60.80**
	GPU	122.67	84.06	78.43	49.24	92.05	58.79	57.74	50.87	49.44	77.70	63.86	66.05	59.75	82.78	64.28	70.51

## Data Availability

The FPDD is available at https://drive.google.com/file/d/1aua3Q2bLpLQKqZ5KTQTHxA5HYoy6U90w/view?usp=sharing (accessed on 27 March 2023) and https://pan.baidu.com/s/1fTkbb2HB_imtgqAegfzJKg?pwd=fpdd (accessed on 14 March 2023).
